# Inhibition of Skin Pathogenic Bacteria, Antioxidant and Anti-Inflammatory Activity of Royal Jelly from Northern Thailand

**DOI:** 10.3390/molecules28030996

**Published:** 2023-01-19

**Authors:** Vitchayaporn Uthaibutra, Thida Kaewkod, Pichet Prapawilai, Hataichanok Pandith, Yingmanee Tragoolpua

**Affiliations:** 1 Department of Biology, Faculty of Science, Chiang Mai University, Chiang Mai 50200, Thailand; 2 Master of Science Program in Applied Microbiology (International Program), Faculty of Science, Chiang Mai University, Chiang Mai 50200, Thailand; 3 Graduate School, Chiang Mai University, Chiang Mai 50200, Thailand; 4 Center of Excellence in Materials Science and Technology, Chiang Mai University, Chiang Mai 50200, Thailand; 5 Research Center in Bioresource for Agriculture, Industry, and Medicine, Faculty of Science, Chiang Mai University, Chiang Mai 50200, Thailand

**Keywords:** antibacterial activity, anti-inflammatory, antioxidant activity, royal jelly, skin pathogenic bacteria

## Abstract

Royal jelly is a nutritious substance produced by the hypopharyngeal and mandibular glands of honeybees. Royal jelly possesses many attractive and beneficial properties which make it an ideal component in medical and pharmaceutical products. The antibacterial, antioxidant, and anti-inflammatory activities of royal jelly from honeybees (*Apis mellifera*) were determined in this study. Moreover, the total phenolic and flavonoid contents of the royal jelly were also evaluated. The effects of royal jelly on growth inhibition against skin pathogenic bacteria, including *Cutibacterium acnes*, methicillin-resistant *Staphylococcus aureus* (MRSA), *Pseudomonas aeruginosa*, *Staphylococcus aureus*, *Staphylococcus epidermidis*, and *Corynebacterium* spp., were investigated by the agar well diffusion method. The minimum inhibitory concentration (MIC) and minimum bactericidal concentration (MBC) were further determined by the broth dilution method. The results indicated that royal jelly showed antibacterial activity by inhibiting the growth of Gram-positive pathogenic bacteria, while the effectiveness decreased against Gram-negative bacteria. Interestingly, royal jelly from Lamphun (RJ-LP1), and Chiang Mai (RJ-CM1), presented high inhibitory efficacy against *C. acnes*, MRSA, and *S. aureus* within 4 h by a time killing assay. Furthermore, the anti-inflammatory properties of royal jelly were tested using RAW264.7 macrophage cells, and results revealed that RJ-LP1 and RJ-CM1 could reduce nitric oxide (NO) production and suppress *iNOS* gene expression. After testing the antioxidant activity, RJ-CM1 and RJ-CM2 of royal jelly from Chiang Mai had the highest level. Additionally, RJ-CM1 also showed the highest total phenolic and flavonoid content. These findings have brought forward new knowledge of the antibacterial, antioxidant, and anti-inflammatory properties of royal jelly, which will improve clinical and pharmaceutical uses of royal jelly as an alternative therapy for bacterial infections, and also as a dietary supplement product.

## 1. Introduction

The skin is the largest organ of the human body, and is located between the external environment and host tissues. It has a rich community of microorganisms and a wide variation of skin microbiota, which depends on the individual’s age, skin site, and time of analysis [1]. As humans mature, physiological changes occur in the skin, leading to significant shifts in the microbiota [2]. The skin plays an important role in the maintenance of physical and immunological barriers by supporting the growth of commensal bacteria, which protects the host from pathogenic bacteria, responsible for cutaneous infections [3]. Cutaneous infections are intricately related to environmental and local factors, host immunity, and organism adherence and virulence. [4].

Under healthy conditions, commensal microbiota on the skin can be beneficial to humans by aiding in nutrition, outcompeting pathogens, and enhancing the immune system [5]. Microbial infections are defended by the human immune system, but if the immune system is impaired, pathogenic bacteria can adhere to host cells, invade, and cause diseases. Bacteria also possess numerous virulent genes that allow bacterial growth in unfavorable environments. Resident bacteria such as *Corynebacterium* spp., *Staphylococcus aureus*, *Staphylococcus epidermidis* and *Pseudomonas aeruginosa* can also cause skin infections [4]. These bacteria can cause diseases by accessing tissue via wounds, for example, *Cutibacterium acnes* (formerly *Propionibacterium acnes*) is the cause of acne. Methicillin-resistant *S. aureus* (MRSA) is a major cause of infection. It contains the *mecA* gene that encodes penicillin-binding protein 2a (PBP-2a), which can reduce the affinity of PBP to β-lactamase antibiotics [6]. 

Free radicals are atoms and molecules with unpaired electrons. They are unstable and highly reactive, and can donate or receive hydrogen atoms or electrons from other molecules. They can lead to oxidative chain reactions which are harmful to the cells of microbes, and are linked to oxidative stress, also resulting in disease. Antioxidant molecules can neutralize free radicals due to the redox potential, which allows them to act as hydrogen donors, reducing agents, and singlet oxygen quenchers. Therefore, they can prevent or delay oxidation reactions by inhibiting the oxidative chain reactions of other molecules [7]. Free radicals may attack soluble cell compounds as well as membranes, eventually leading to the impairment of cell functions and cytolysis [8].

Nowadays, modern medicine is facing serious problems because of the side-effects of synthetic drugs and an increase in antibiotic-resistant microorganisms. Because of this, the growing rate of severe diseases has pushed for wider research of natural products for safer therapy [9]. Apitherapy is a form of alternative medicine that uses the bioactive properties of bee products to prevent and/or treat different diseases [10]. Honeybees are important to humans and are gaining more interest due to their health-promoting assets. Historically, bee products have been used for therapeutic purposes, nutritional properties, and biological activities for many years [9]. Humanity has benefited from bee products over the centuries for treating and preventing various illnesses, and apitherapy has been employed in several countries as a complementary medicine [11].

Royal jelly is a white-yellowish viscous substance, with a sour and sweet taste and a slightly pungent odor of phenol, which is secreted by the hypopharyngeal and mandibular glands of young worker bees (*Apis mellifera*) [12]. It is used to feed developing larvae during the first 3 days of life, and to feed the queen bee for her entire lifespan. Royal jelly has a significant impact on bee lifespan. Worker bees live for around 45 days, while the queen bee can live for up to 5 years. Royal jelly contains amino acids, carbohydrates, lipids, minerals, vitamins, and the bioactive compound trans-10-hydroxy-2-decenoic acid (10-HDA) [13,14,15]. The composition of royal jelly is affected by various factors, such as the seasons, collector type, floral variety, time of harvest, and geographical and environmental conditions [16,17]. Many beneficial properties of royal jelly have been observed, such as anti-inflammatory [16,18], antioxidative [16,17,18], antimicrobial [16,18], anti-tumoral [16,18,19], immune activating [18], and wound healing activities [18]. The bioactive proteins of royal jelly are known to have immune regulatory and antibacterial effects from several studies [20]. In honeybees, these proteins may be involved in an active defense system against bacterial infection, and royal jelly is believed to exert similar effects in humans. [16].

In recent years, the use of royal jelly has increased due to its attractive properties and health benefits as a natural bee product, and its great potential for application in medical and pharmaceutical products [21,22]. Generally, it is used in cosmetics, health food, food supplements, medical products and pharmaceutical products [15,23]. This research evaluates the antibacterial, antioxidant, and anti-inflammatory activities, as well as the total phenolic and flavonoid content, of royal jelly, in order to improve knowledge for its potential use as an alternative therapy in the future.

## 2. Results

### 2.1. Antibacterial Activity of Royal Jelly

The antibacterial activity of the royal jelly samples on skin pathogenic bacteria was determined using the agar well diffusion method, by measuring the diameter of the inhibition zone. The results showed that royal jelly at 300 mg/mL exhibited antibacterial activity against skin pathogenic bacteria, including *Cutibacterium acnes*, methicillin-resistant *Staphylococcus aureus* (MRSA), *Pseudomonas aeruginosa*, *Staphylococcus aureus*, *Staphylococcus epidermidis* and *Corynebacterium* spp., with the inhibition zone ranging from 9.33 ± 0.58 to 27.67 ± 2.31 mm (Table 1). Royal jelly samples from Lamphun province (RJ-LP1), and from Chiang Mai province (RJ-CM2, RJ-CM3 and RJ-CM4), were able to inhibit all tested bacteria, whereas RJ-CM1 and RJ-CM7 inhibited all tested bacteria except *P. aeruginosa*.

### 2.2. Minimum Inhibitory Concentration (MIC) and Minimum Bactericidal Concentration (MBC) of Royal Jelly 

The minimum inhibitory concentration (MIC) and minimum bactericidal concentration (MBC) of the royal jelly samples were determined by the broth dilution method. The results for the MIC and MBC are reported in Table 2. Royal jelly had MIC and MBC values ranging from 18.75 to 150.00 mg/mL against skin pathogenic bacteria. All samples had the lowest MIC and MBC values on *Corynebacterium* spp. In addition, royal jelly samples RJ-LP1, RJ-CM1, RJ-CM2, RJ-CM4, and RJ-CM5 were effective against skin pathogenic bacteria such as *C. acnes*, MRSA, *P. aeruginosa* and *S. aureus*, with MBC values in the range of 37.50 to 75.00 mg/mL. The royal jelly samples were tested and compared with a negative control: distilled water and antibiotic drugs. The positive control was gentamicin and vancomycin (for MRSA).

### 2.3. Time Killing Assay of Royal Jelly 

**Figure 1:**
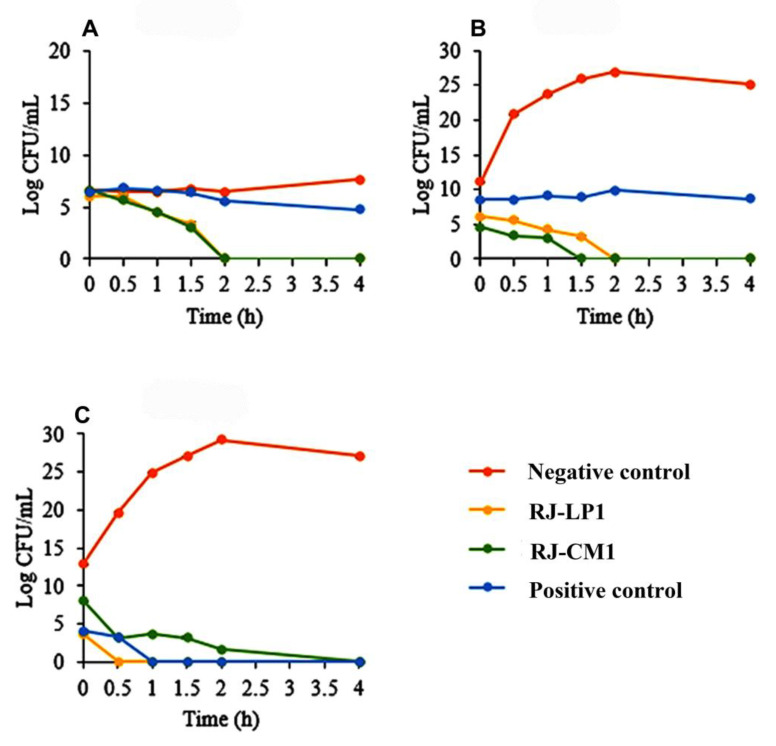
Time killing curve of royal jelly samples RJ-LP1 and RJ-CM1 on *C. acnes* (**A**), MRSA (**B**) and *S. aureus* (**C**). Time-kill data represented as mean ± SD (*n* = 3). The negative control contained the medium without royal jelly. Gentamicin at 0.0039 mg/mL and 0.0156 mg/mL was used as the positive control for *C. acnes* and *S. aureus*, respectively. Vancomycin at 0.0020 mg/mL was used as the positive control for MRSA.

Royal jelly samples RJ-LP1 and RJ-CM1, at MBC concentrations, were selected and determined for time killing on *C. acnes*, MRSA, and *S. aureus*. RJ-LP1 was tested at 37.50, 75.00 and 75.00 mg/mL, while RJ-CM1 was tested at 37.50, 75.00 and 75.00 mg/mL and incubated with *C. acnes*, MRSA and *S. aureus*, respectively. Bacterial growth was quantified at 0, 0.5, 1, 1.5, 2, 4, 6, 8, 10, 12 and 24 h after being treated with the royal jelly. RJ-LP1 inhibited *C. acnes* and MRSA by 50% at 0 h and 1.5 h, respectively, and 100% at 2 h. *S. aureus* was completely inhibited after 0.5 h of incubation. RJ-CM1 inhibited *C. acnes* at 1.5 h and 2 h, by 50% and 100%, respectively, while MRSA and *S. aureus* were completely inhibited at 1.5 h and 4 h of incubation, respectively (Figure 1).

### 2.4. Anti-Inflammatory Activity

#### 2.4.1. Cytotoxicity of Royal Jelly on RAW264.7 Cells

The cytotoxic effect of the royal jelly samples on RAW264.7 macrophage cells was evaluated by an MTT assay. The treatment was tested and compared to the cell control group (CC) and vehicle control group (VC), which was DI water. The results of cell viability after 24 h of incubation using royal jelly powder and fresh royal jelly, at concentrations of 40 and 60 mg/mL, respectively, are as follows (Figure S1). The cell viability decreased, while the royal jelly concentration increased. Results indicated that royal jelly powder at a concentration of ≤20 mg/mL, and fresh royal jelly at ≤30 mg/mL, did not significantly affect cell viability. Therefore, royal jelly at non-cytotoxic concentrations was selected for the subsequent experiments.

#### 2.4.2. Inhibitory Effects of Royal Jelly on Nitric Oxide (NO) Production 

**Figure 2:**
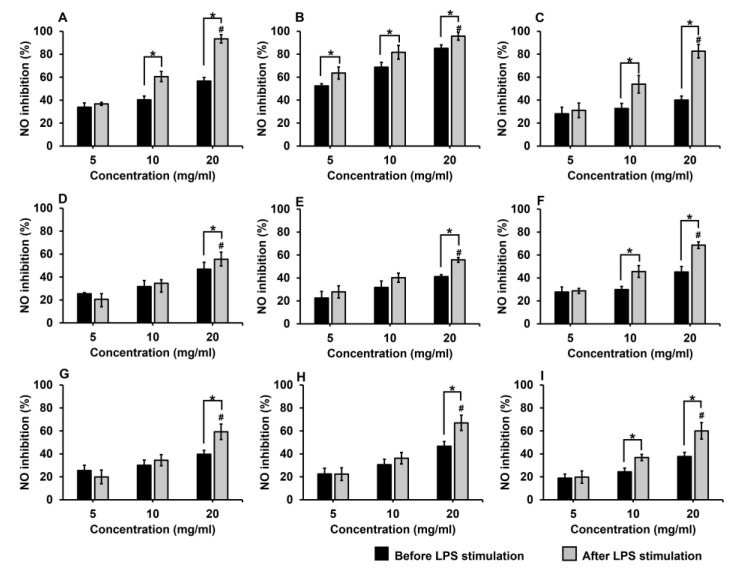
Inhibitory effects of royal jelly samples RJ-LP1 (**A**) and RJ-CM1–RJ-CM8 (**B–I**) on NO production when treated before and after stimulation of RAW264.7 cells with LPS for 24 h. The data represents mean values of three replicates ± SD. # The significant highest NO inhibition is shown in each sample (*p* < 0.05). * The values were significantly different before and after stimulation with LPS of RAW264.7 cells (*p* < 0.05).

The effect of the royal jelly samples on NO production was estimated using the Griess reaction. Royal jelly samples at varied concentrations were applied to RAW264.7 cells induced by lipopolysaccharides (LPS). After 24 h of incubation, the nitrite content present in the culture supernatants was determined as an indirect measurement of NO production. The results showed that the NO production was reduced in a dose-dependent manner when the cells were treated with various concentrations of royal jelly (5–20 mg/mL). Moreover, adding the royal jelly after stimulation with LPS could inhibit NO production more effectively than adding royal jelly before stimulation with LPS (Figure 2). 

Royal jelly at a concentration of 20 mg/mL could inhibit the NO production before stimulation with LPS by 37.73–85.12%, and after stimulation with LPS by 55.47–95.69%. The results revealed that royal jelly sample RJ-CM1 had the highest percentage of NO inhibition both before and after stimulation with LPS, with values of 85.12% and 95.69%, respectively. Moreover, the half maximal inhibitory concentration (IC_50_) was calculated, and revealed that RJ-CM1 was the most effective to inhibit NO production both before and after stimulation with LPS, with the IC_50_ of 5.83 mg/mL and 4.46 mg/mL, respectively. Subsequently, royal jelly sample RJ-LP1 showed the IC_50_ of 15.99 mg/mL and 9.23 mg/mL, to inhibit NO production both before and after stimulation with LPS, respectively (Table 3). 

#### 2.4.3. Inhibitory Efficacy of Royal Jelly on Inflammatory Gene Expression

**Figure 3:**
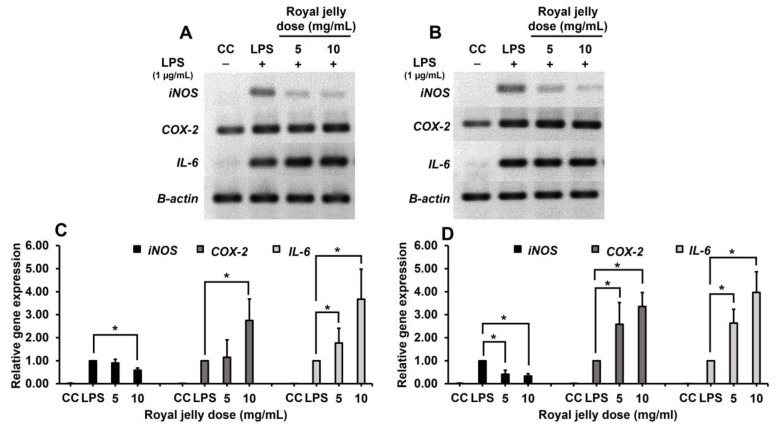
The inflammatory gene expression of RAW264.7 cells after treatment with royal jelly samples RJ-LP1 (**A**,**C**) and RJ-CM1 (**B**,**D**) by agarose gel electrophoresis (**A**,**B**) and qRT-PCR (**C**,**D**). The genes were detected with specific primers (*iNOS, COX-2* and *IL-6*). The data represent mean values of three replicates ± SD. * Royal jelly concentrations were significantly different when compared with LPS control (*p* < 0.05).

The inhibitory effect of royal jelly samples RJ-LP1 and RJ-CM1 on inflammatory gene expression of RAW264.7 cells, including *iNOS, COX-2* and *IL-6,* was analyzed by the RT-PCR method. Total RNA was extracted from the RAW264.7 cells stimulated with LPS after treatment with royal jelly for 3 h. Then RNA was reversed into cDNA by the reverse transcriptase enzyme. The target genes were detected by specific primers, and the β-actin gene was used as an internal control. PCR products were investigated by agarose gel electrophoresis, and visualized by the gel documentation system. Furthermore, the level of mRNA gene expression was also evaluated by the qRT-PCR method. The results showed that LPS could induce the expression of inflammatory genes, which showed the upregulated expression compared with a cell control group. On the other hand, the treatment of the RAW264.7 cells with royal jelly samples RJ-LP1 and RJ-CM1 at concentrations of 5 and 10 mg/mL for 3 h could suppress the expression of the *iNOS* gene in a dose-dependent manner. However, both samples could not inhibit the expression of *COX-2* and *IL-6* genes. The results also indicated that royal jelly did not affect the expression of a housekeeping gene (β-actin) (Figure 3).

### 2.5. Antioxidant Activity of Royal Jelly

The antioxidant activity of the royal jelly samples was determined by an ABTS assay and the 50% inhibitory concentration (IC_50_) values were calculated. Royal jelly showed antioxidant activity in the range of 0.89 ± 0.14 to 4.13 ± 1.89 mg TEAC/g royal jelly. Royal jelly samples RJ-CM1 and RJ-CM2 exhibited the highest antioxidant activity, with values of 4.13 ± 1.89 and 3.61 ± 0.47 mg TEAC/g royal jelly, respectively (Table 4).

### 2.6. Total Phenolic Content of Royal Jelly 

The total phenolic compound contents of the royal jelly samples are shown in Table 4. The values vary in the range of 1.82 ± 0.38 to 8.61 ± 1.57 mg GAE/g royal jelly. The highest total phenolic content was found from royal jelly sample RJ-CM1, followed by RJ-CM2 and RJ-LP1, with values of 8.61 ± 1.57, 7.27 ± 2.99 and 6.64 ± 1.88 mg GAE/g royal jelly, respectively.

### 2.7. Total Flavonoid Content of Royal Jelly

The total flavonoid contents of the royal jelly samples are shown in Table 4. The values vary in the range of 0.28 ± 0.09 to 6.25 ± 0.59 mg QUE/g royal jelly. Royal jelly sample RJ-CM1 exhibited the highest total flavonoid content, 6.25 ± 0.59 mg QUE/g royal jelly. Subsequently, royal jelly samples RJ-CM2 and RJ-LP1 had a flavonoid content of 5.13 ± 0.86 and 4.93 ± 0.45 mg QUE/g royal jelly, respectively.

### 2.8. HPLC Analysis of Phytochemical Content of Royal Jelly

**Figure 4:**
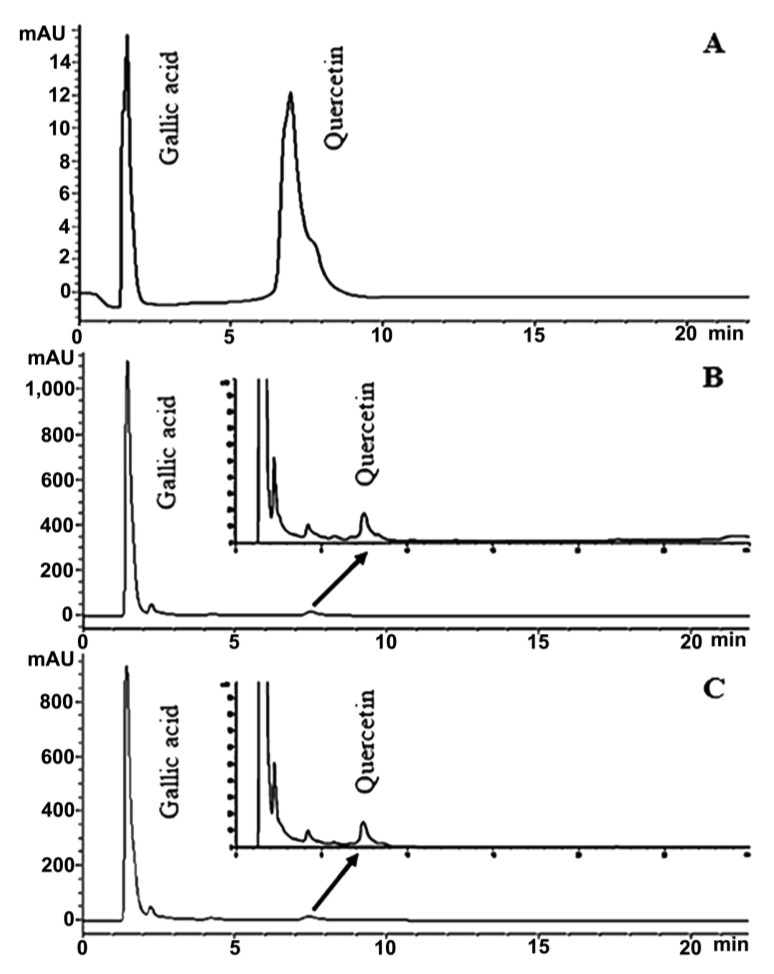
HPLC chromatogram of gallic acid and quercetin (**A**) found in royal jelly sample RJ-LP1 (**B**) and RJ-CM1 (**C**).

Phytochemical compounds including gallic acid and quercetin in royal jelly samples RJ-LP1 and RJ-CM1 were analyzed using the HPLC method. RJ-LP1 and RJ-CM1 showed a gallic acid content of 6.68 ± 0.60 and 6.50 ± 0.83 mg gallic acid/g royal jelly, respectively. They also showed quercetin content in values of 0.26 ± 0.01 and 0.24 ± 0.03 mg quercetin/g royal jelly, respectively (Figure 4).

## 3. Discussion

Royal jelly has broad-spectrum antimicrobial activity against microorganisms including bacteria, fungi, yeasts, and viruses. It has been reported that antibacterial activity of royal jelly is due to the fatty acids present, especially 10-HDA. Moreover, the short peptides jelleines and royalisin also showed strong antibacterial properties [24]. Royalisin and jelleines were able to decrease bacterial cell hydrophobicity and disrupt cell membrane permeability in certain Gram-positive bacteria, leading to disruption and dysfunction of the bacterial cell walls and cell membranes [25,26]. The major royal jelly proteins (MRJPs) and royalactin are the main sources of antibacterial activity in royal jelly, especially against Gram-positive bacteria. [15].

In this study, royal jelly was tested for antibacterial activity against skin pathogenic bacteria. Gentamicin was used as a positive control because of its broad-spectrum activity. Vancomycin was also used as a positive control for MRSA. The MIC and zone diameter breakpoint for methicillin-resistant Staphylococci were >4 mg/L and ≤19 mm, respectively [27]. The results by agar well diffusion showed that royal jelly samples RJ-LP1, RJ-CM2, RJ-CM3, and RJ-CM4 could inhibit all tested bacteria, whereas RJ-CM1 and RJ-CM7 could inhibit all tested bacteria except *P. aeruginosa*. The results of the MIC and MBC indicated that all royal jelly samples showed antibacterial activity against tested bacteria, with values ranging from 18.75 mg/mL to 150 mg/mL. In addition, royal jelly samples RJ-LP1 and RJ-CM1, at MBC concentrations, were selected and evaluated for time killing on *C. acnes*, MRSA and *S. aureus,* and exhibited high activity compared to antibiotic drugs. According to the results, some royal jelly samples did not show an inhibition zone using the agar well diffusion method, but still showed activity against the tested bacteria by the broth dilution method. This may be because royal jelly has a high viscosity that may cause low diffusion in agar. Additionally, the thickness of agar might affect the competence of royal jelly’s antibacterial activity. However, in the MIC and MBC methods, the samples that contacted the bacterial cells directly proved to be more effective. Osés et al. (2016) expressed that the agar well diffusion method has been described as a method with relatively low sensitivity, since the samples were further diluted as they diffused into the agar [28]. Thus, they suggested that the agar well diffusion method should be combined with other procedures such as the broth dilution method.

The overall results of this study showed that Gram-positive bacteria were more sensitive to royal jelly than Gram-negative bacteria. Similarly, many researchers have reported that royal jelly showed higher antibacterial activity against Gram-positive bacteria than against Gram-negative bacteria [29,30]. Antibacterial peptides from royal jelly are positively charged due to the presence of arginine, histidine, and lysine, which allows them to interact with anionic phospholipids of the cell membrane, and cause cell membrane disruption [31]. In addition, royalisin is a member of insect defensins, which can bind to the surface of microbial membranes and cause lysis of the intracellular contents. Royalisin forms voltage-dependent channels in bacteria, then disrupts the permeability barrier of the cytoplasmic membrane, leading to the loss of cytoplasmic potassium, partial depolarization of the inner membrane, a decrease in cytoplasmic ATP, and respiratory inhibition [32]. Previous studies also indicated that royalisin has the ability to decrease bacterial cell hydrophobicity and disrupt cell membrane permeability in certain Gram-positive bacteria, leading to disruption and dysfunction of the bacterial cell walls and cell membranes [25]. Furthermore, the presence of an outer membrane consisting of lipopolysaccharides in Gram-negative bacteria could limit the permeability of hydrophobic molecules. The complex structure of the outer membrane can also protect the cells from chemicals and enzymes. On the other hand, Gram-positive bacteria consist of a mostly peptidoglycan layer, that is not an effective barrier of permeability [33,34].

The antioxidant activity present in royal jelly may derive from phenolic compounds, amino acids, carboxylic fatty acids, and vitamins [23]. Royal jelly contains phenolic compounds that worker bees collect from plants, where they gather nectar [35,36]. The main phenolic compound groups found in plants are derivatives of the flavonoids, cinnamic acid and cumarins [37]. Phenolic compounds and polyphenols have been reported to show anti-inflammatory, anti-carcinogenic, and analgesic activities, and immune modulation, and exert these functions as antioxidants [29]. These antioxidant properties, found in phenolic and flavonoid compounds, play an important role in the pharmacological uses of royal jelly. 10-HDA is also known to possess various biological properties, including antioxidant activity [37,38]. Park et al. (2019) also reported that the major royal jelly proteins (MRJP) in royal jelly showed antimicrobial and antioxidant activities [39].

In this study, the antioxidant activity of the royal jelly samples was determined by an ABTS assay. The total phenolic, flavonoid, and phytochemical content of the samples was analyzed by a Folin-Ciocalteu assay, aluminum chloride colorimetric assay, and by an HPLC method, respectively. The overall results showed that royal jelly samples RJ-CM1 and RJ-CM2 exhibited the highest antioxidant activity. Royal jelly sample RJ-CM1 had the highest total phenolic and flavonoid content, which may be the major component responsible for antioxidant activity in royal jelly via redox properties [40]. Moreover, the results of the HPLC method showed that royal jelly samples RJ-LP1 and RJ-CM1 had gallic acid contents of 6.68 ± 0.60 and 6.50 ± 0.83 mg gallic acid/g royal jelly, respectively. They also revealed quercetin contents with values of 0.26 ± 0.01 and 0.24 ± 0.03 mg quercetin/g royal jelly, respectively. The gallic acid content may be relative to the total phenolic content in the samples, since it was one of the phenolic derivatives. Due to the low content of flavonoids in the samples, quercetin may also have been present in low quantities, or none at all. Since this study used only quercetin as a standard for the analysis, the results may not reflect the total flavonoid content, since there are many types of flavonoids and derivatives in royal jelly, rather than only quercetin. The principal flavonoids present in royal jelly include flavonoles (e.g., quercetin, kaempherol, galangin, and fisetin), flavanones (e.g., pinocembrin, naringin, and hesperidin), and flavones (e.g., apigenin, acacetin, chrysin, and luteolin) [18]. However, the composition of royal jelly and its antioxidant capacity is influenced by various factors such as the environment, flower nectar sources, time of harvest, seasons, and processing methods [10].

The results of this study show that royal jelly has a strong antioxidant activity due to the short peptides that exhibit hydroxyl radical scavenging, metal chelating, and superoxide anion radical scavenging activities. These peptides contain tyrosine residues at the C-terminal, which allow strong hydroxyl radical and H_2_O_2_ scavenging activity. In addition, 10-HDA and other fatty acids in royal jelly have been reported to exhibit radical scavenging activity [21,23]. The antioxidative activity of royal jelly has been studied in previous experiments. The results showed that royal jelly and other bee products have high antioxidant activity [7,41]. They also suggested that organic acids in royal jelly contributed to the antioxidant activity via metal chelation, and increased the effect of total polyphenols. In addition, the findings of Park et al. (2020) indicated that MRJPs 1–7 function as important constituents in the antioxidant capacity of royal jelly, through a protective role against oxidative stress-induced cell apoptosis, and could increase cell viability [42]. Furthermore, MRJPs 1–7 could provide DNA protection against ROS. The protection against oxidative stress and lipid peroxidation have been confirmed in experiments on laboratory animals. After feeding royal jelly to mice for 16 weeks, the levels of 8-hydroxy-2-deoxyguanosine (an oxidative stress marker) in kidney DNA and serum were significantly reduced, and the average life span of C3H/HeJ mice increased through the mechanism of reduced oxidative damage [43].

Inflammation is an important host response, which is prompted by stimulations such as infections, chemical toxins, mechanical injuries, and many other reactions [44]. In bacterial infection in humans, the host cells are stimulated by bacteria, and produce pro-inflammatory cytokines. However, the over-production of cytokines may lead to chronic inflammation [45]. The use of anti-inflammatory drugs can cause serious side effects, therefore, this study strived to use natural substances which are a safer alternative [46]. Royal jelly is an important and useful substance that has been incorporated into several products due to its beneficial and therapeutic properties. The biological activities of royal jelly are mainly attributed to fatty acids, proteins, bioactive compounds, and the phenolic and flavonoid content. The lipid composition of royal jelly is comprised of 80–85% fatty acids, combined with proteins (MRJPs) that are responsible for its activities [21,44,47].

The cytotoxic effect of the royal jelly samples on RAW264.7 cells was determined by an MTT assay. The royal jelly samples were tested and compared with the cell control group and vehicle control group (DI water). After 24 h of incubation, the results showed that royal jelly powder and fresh royal jelly at ≤20 and ≤30 mg/mL, respectively, did not significantly affect the cell viability. Then, the non-cytotoxic concentrations were selected to evaluate the nitric oxide (NO) production by the Griess reaction. The results showed that royal jelly was able to inhibit NO production in a dose-dependent manner. Moreover, applying royal jelly after stimulation with LPS had the ability to inhibit NO production in RAW264.7 cells more than applying royal jelly before stimulation with LPS. Regarding the in vitro inflammation in macrophages, LPS significantly induced the production of inflammatory cytokines and mediators, such as NO and tumor necrosis factor-alpha (TNF-α). Interestingly, the properties of royal jelly samples in hydrophilic and lipophilic forms of royal jelly could reduce NO production in RAW264.7 cells, which was similar to royal jelly from *A. mellifera* during the blossom seasons of *Castanea mollissima* Bl. (chestnut) and *Brassica napus* L. (rapeseed) floral sources. Additionally, the hydrophilic extracts presented higher anti-inflammatory efficacy than the lipophilic extracts [48]. Therefore, the composition and activities of royal jelly depend on the honeybee species, environmental conditions, harvest time, and plant sources [49].

Macrophages are innate immune cells that play an important role in the inflammatory response. LPS stimulates toll-like receptor 4 (TLR4) on the cell surface and induces the activation of nuclear factor kappa B (NF-κB) signaling pathways, resulting in the secretion of proinflammatory mediators. TNF-α and interleukin 1-β (IL-1β) could also induce the expression of several mediators, such as prostaglandins, leukotrienes, and NO [50,51]. By promoting the synthesis of prostaglandins, IL-1β increases *COX*-2 transcription. NO is generated by the catalyst of inducible NO synthase (iNOS), and leads to cell growth, proliferation, and differentiation under inflammatory responses. However, a high level of NO could induce cell damage, cancer, and chronic inflammation [52]. Thus, the inhibitory activities of royal jelly on the production of NO in cells are beneficial in protecting the cells from inflammation and providing a wound healing regeneration process [48].

In this study, the effects of royal jelly samples RJ-LP1 and RJ-CM1 on anti-inflammatory gene expression, including *iNOS*, *COX*-2, and *IL*-6, were evaluated by gel electrophoresis and the qRT-PCR method. The results showed that both samples could reduce transcription of the *iNOS* gene. On the other hand, they could not downregulate the expression of the *COX*-2 and *IL-6* genes. The anti-inflammatory effects of royal jelly correlated with the main fatty acids in royal jelly such as trans-10-hydroxy-2-decenoic acid (10-H2DA), 10-hydroxydecanoic acid (10-HDAA), and sebacic acid (SEA), which significantly inhibited NO production and *iNOS* expression by blocking the phosphorylation of ERK1/2, JNK1/2 and mediating JNK signaling pathways, respectively [44]. Moreover, the study also reported that 10-H2DA showed modest attenuating effects on I𝜅B𝛼, and enhanced the phosphorylation of p65, suggesting that 10-H2DA regulated LPS stimulation through more than one single pathway. 10-HDAA showed no significant effects on the NF-𝜅B pathway, whereas SEA exerted a strong suppression on p65 phosphorylation, indicating that SEA inhibited transcription of *iNOS*, *IL-10*, *TNF-𝛼*, and *COX-*2 expression, by suppressing the activity of NF-𝜅B. Takahashi et al. (2013) also reported that *IL-6* and *TNF-α* mRNA expression did not decrease when treated with 10-HDAA for 3, 6, 12, and 24 h [53]. However, Kohno et al. (2004) showed that royal jelly could inhibit pro-inflammatory cytokine production, including TNF-α, IL-1 and IL-6, in a dose-dependent manner, by suppressing the production of prostaglandins E2. They also indicated that royal jelly contains MRJP3, a factor responsible for the suppression of proinflammatory cytokine secretion [54].

## 4. Materials and Methods

### 4.1. Reagents and Chemicals

Brain Heart Infusion (BHI) broth and (BHIA) Mueller–Hinton (MH) broth for antimicrobial susceptibility testing were purchased from BD (Becton, Dickinson and company, Franklin Lakes, NJ, USA). RAW264.7 cell culture media and Dulbecco’s modified Eagle’s medium (DMEM) were purchased from Gibco (Grand Island, NY, USA). The MTT solution for cytotoxicity assay was purchased from Bio Basic (Amherst, NY, USA). Lipopolysaccharide (LPS), N-(1-naphthyl)-ethylenediamine, sulfanilamide, and phosphoric acid for anti-inflammatory activity were obtained from Merck (Billerica, MA, USA) and Sigma-Aldrich (St. Louis, MO, USA). 2,20-azinobis-(3-ethylbenzothiazolin-6 sulfonic acid) (ABTS), potassium persulfate, 6-hydroxy-2,5,7,8-tetramethyl-chroman-2-carboxylic acid (Trolox), and gallic acid monohydrate were purchased from Sigma-Aldrich (St. Louis, MO, USA). Folin–Ciocalteu reagent, TPTZ (2,4,6-tri(2-pyridyl)-s-triazine) and quercetin dehydrate were obtained from Merck (Billerica, MA, USA). Ethanol, methanol, sodium carbonate, aluminum chloride, potassium acetate, and formic acid for total phenolic and flavonoid detection, and HPLC assay were obtained from RCl Labscan (Bangkok, Thailand). All reagents and chemicals used were analytical grade.

### 4.2. Royal Jelly Samples

Three samples of royal jelly powder (RJ-LP1, RJ-CM1, RJ-CM2), and six samples of fresh royal jelly (RJ-CM3-RJ-CM8), were obtained from bee farms in Lamphun and Chiang Mai Provinces, Thailand, and were kindly provided by Assoc. Prof. Dr. Panuwan Chantawannakul, Division of Microbiology, Department of Biology, Faculty of Science, Chiang Mai University and Bee products Industry Co., Ltd, Thailand. The royal jelly samples were collected from bee farms during 2018–2019 (May-September). Royal jelly powders were prepared by lyophilization, and fresh royal jelly samples were kept in amber bottles to protect them from light, and were stored at 4 °C and −20 °C, respectively. All samples were dissolved in distilled water before use.

### 4.3. Bacterial Strains and Culture

Skin pathogenic bacteria, including methicillin-resistant *Staphylococcus aureus* (MRSA) 49, *Pseudomonas aeruginosa* ATCC 27,853, *S. aureus* ATCC 25,923, *S. epidermidis* ATCC 14,990 and *Corynebacterium* spp., were cultured on Mueller–Hinton agar (MHA) at 37 °C for 18–24 h. *Cutibacterium acnes* DMST 14,916 was cultured on Brain Heart Infusion agar (BHIA) at 37 °C for 48–72 h in anaerobic conditions. All tested bacteria were obtained from the Division of Microbiology, Department of Biology, Faculty of Science, Chiang Mai University, Chiang Mai, Thailand.

### 4.4. Antibacterial Activity by Agar Well Diffusion Assay

The effects of royal jelly were investigated for growth inhibition against skin pathogenic bacteria by the agar well diffusion method. Bacterial cultures were adjusted to McFarland standards No. 0.5 (1 × 10^8^ CFU/mL) and swabbed on agar plates. The agar plates were then punched aseptically with a 6–8 mm diameter sterile cork borer. Next, 100 µL of royal jelly (300 mg/mL) and controls were added to the wells. Gentamicin and vancomycin were used at 1 mg/mL as the positive control, and distilled water was applied as the negative control. Gentamicin was used for all tested bacteria except MRSA, while vancomycin was used for MRSA. The agar plates were then incubated under suitable conditions depending upon the tested microorganisms. After that, the diameters of the inhibition zones were determined. Each experiment was performed independently in triplicate [55].

### 4.5. Minimum Inhibitory Concentration (MIC) and Minimum Bactericidal Concentration (MBC) Analysis

The MIC values of the royal jelly samples were evaluated by the broth dilution method. Royal jelly (300 mg/mL) and antibiotic drug positive controls (1 mg/mL) were prepared by serial two-fold dilutions in broth and added to test tubes. Then the microbial inoculum was inoculated after being diluted to standardized microbial suspension, McFarland No. 0.5. After well-mixing, the inoculated tubes were incubated under suitable conditions depending upon the tested microorganisms. The tubes were then examined for growth or turbidity. The MBC was determined by streak plate from tubes from which there was no visible bacterial growth, and the agar plates were incubated under suitable conditions depending upon the tested microorganisms. The MBC endpoint was defined as the lowest concentration of royal jelly that killed more than 99.9% of bacteria [55].

### 4.6. Time Killing Assay

The time-dependent antimicrobial effect was determined by a time-kill assay. Bacterial growth was quantified at 0, 0.5, 1, 1.5, 2, 4, 6, 8, 10, 12, and 24 h after treatment with royal jelly. Royal jelly samples at the MBC concentration were mixed with bacteria inoculum at a ratio of 1:1 and incubated under suitable conditions depending upon the tested bacteria. The mixture was then diluted with 0.85% NaCl and dropped on agar plates for 20 µL. A control test was performed for the bacteria without royal jelly or antibiotic, and the colony forming unit (CFU) of the bacteria after treatment with royal jelly was determined. A graph of the log CFU/mL of bacteria was plotted against the time of royal jelly treatment [56].

### 4.7. Cytotoxicity Assay of Royal Jelly on RAW264.7 Cells

RAW264.7 cells were cultured in Dulbecco’s modified Eagle’s medium (DMEM) supplemented with 10% fetal bovine serum (FBS), penicillin (100 units/mL), and streptomycin (100 µg/mL). The cell culture was maintained in a humidified atmosphere in a 5% CO_2_ incubator for 48 h. The RAW264.7 cells were seeded in a 96-well plate (1 × 10^6^ cell/well) and kept at 37 °C in a humidified atmosphere with a 5% CO_2_ incubator for 24 h. The cells were then treated with various concentrations of royal jelly after dilution with serum-free DMEM. After treatment for 24 h, cell viability was measured by MTT (3-(4,5-dimethylthiazol-2-yl)-2, 5-diphenyl tetrazolium bromide) assay. Thirty microliters of MTT reagent (2 mg/mL) were added into each well and incubated for 4 h. Next, 200 µL of DMSO was added to solubilize the formazan crystals and was incubated for 10 min in dark conditions. The absorbance was measured at 540 nm, with a reference wavelength of 630 nm, by microplate reader [57].

### 4.8. Anti-inflammatory Activity of Royal Jelly

#### 4.8.1. Treatment of RAW264.7 Cells by Royal Jelly before Stimulation by Lipopolysaccharide

RAW264.7 cells were seeded in a 96-well plate (1 × 10^6^ cell/well) and cultured at 37 °C in a humidified atmosphere with a 5% CO_2_ incubator for 24 h. The media was then removed and replaced with 100 µL of royal jelly at various concentrations. After an hour of exposure, the royal jelly was removed and LPS (1 µg/mL) was added to each well. After incubation for 24 h, 100 µL of supernatant was moved into a new 96-well plate and mixed with 100 µL of Griess reagent (0.1% (*w*/*v*) N-(1-naphthyl)-ethylenediamine and 1% (*w*/*v*) sulfanilamide in 5% (*v*/*v*) phosphoric acid). The absorbance was measured at 540 nm using a microplate reader [48].

#### 4.8.2. Treatment of RAW264.7 Cells by Royal Jelly after Stimulation by Lipopolysaccharide

RAW264.7 cells were seeded in a 96-well plate (1 × 10^6^ cell/well) and cultured at 37 °C in a humidified atmosphere with a 5% CO_2_ incubator for 24 h. The cells were then stimulated with LPS (50 µL) and incubated for 10 min. After that, LPS was removed and the royal jelly at various concentrations was added to each well. After incubation for 24 h., 100 µL of supernatant was moved into a new 96-well plate and mixed with 100 µL of Griess reagent. The absorbance was measured at 540 nm using a microplate reader.

#### 4.8.3. Measurement of Nitric Oxide (NO) Production

The production of NO was determined based on the presence of nitrite in the supernatant using Griess reagent. RAW264.7 cells were stimulated by lipopolysaccharide (LPS). LPS and royal jelly were diluted with DMEM without phenol red. After that, the nitric oxide production from royal jelly treated cells and non-treated cells were measured by Griess assay [57]. The absorbance was measured at 540 nm. The nitrite concentration was determined by a standard curve of sodium nitrite present in DMEM without phenol red. Three independent experiments with three replicates at each concentration were conducted.

### 4.9. Inflammatory Gene Expression by Quantitative Real-time Polymerase Chain Reaction (qRT-PCR)

RAW264.7 cells were seeded in a 24-well plate (5 × 10^5^ cell/well) and cultured at 37 °C in a humidified atmosphere with a 5% CO_2_ incubator for 24 h. Next, the cells were stimulated by LPS (1 µg/mL) and treated with royal jelly at various concentrations. After 3 h, total RNA was isolated from the cells by NucleoSpin^®^ RNA (Macherey-Nagel, Nordrhein-Westfalen, Germany). The quality and quantity of RNA were measured with a Nanodrop spectrophotometer. One microgram of RNA was reverse transcribed using ReverTra Ace^®^ (TOYOBO, Osaka, Japan) to obtain cDNA according to the manufacturer’s protocol. The master mix of the reaction contained 100 ng of cDNA, 2X SensiFAST SYBR^®^ No-ROX Mix (BIOLINE, London, UK), 400 nM of the forward primer, and 400 nM of the reverse primer. PCR conditions were applied as follows: denaturation was performed at 95 °C for 2 min; amplification and quantification were repeated for 40 cycles at 95 °C for 5 secs; and 60 °C for 30 secs, cycle threshold (Ct) values of the inflammatory genes, including *iNOS, COX-2* and *IL-6,* were calculated from the relative expression by normalizing the data with the expression of the internal control gene (*β-actin*); PCR products were investigated by agarose gel electrophoresis and visualized by gel documentation; sequences of the forward and reverse primers for PCR were designed based on previous studies [58,59,60]. Primer sequences for the amplification of apoptotic genes are shown in Table S1. 

### 4.10. Antioxidant Activity of Royal Jelly by ABTS Radical Cation Decolorization Assay

The antioxidant activity of royal jelly was determined by an ABTS (2, 2’-azino-bis (3-ethylbenzothiazoline-6-sulphonic acid)) radical cation decolorization assay. To produce the ABTS radical cation (ABTS^•+^) mixture, 7 mM ABTS was mixed with 2.45 mM potassium persulfate (K_2_S_2_O_8_). The mixture was kept at room temperature in dark conditions for 12–16 h to reach a stable oxidative state. The working solution was prepared by diluting the mixture with DI water to achieve an absorbance of 0.700 ± 0.020 at 734 nm. Royal jelly was mixed with the ABTS^•+^ solution and incubated in dark conditions for 10 min. After that, the absorbance was measured at a wavelength of 734 nm by a microplate reader (Biochrom, UK). Antioxidant activity is determined as the ability to scavenge 50% of free radicals (IC_50_) and is expressed as Trolox equivalent antioxidant capacity per gram royal jelly (TEAC/g royal jelly) [61].

### 4.11. Total Phenolic Compound Analysis

The total polyphenol content of royal jelly was evaluated by the Folin–Ciocalteu method, according to the protocol of Singleton et al., (1999) with slight modifications using gallic acid as a calibration standard [62]. Royal jelly (250 µL) was mixed with 125 µL of 50% Folin–Ciocalteu reagent, 1.25 mL of DI water, and 250 µL of 95% ethanol. The mixture was then incubated in dark conditions for 5 min. After that, 250 µL of 5% sodium carbonate was added and incubated in dark conditions for 1 h. Next, the absorbance was measured at 765 nm by a spectrophotometer (Thermo Scientific, USA). The total phenolic content was calculated and reported as milligrams of gallic acid equivalent per gram of royal jelly (mg GAE/g royal jelly).

### 4.12. Total Flavonoid Compound Analysis

The total flavonoid content of royal jelly was evaluated by the aluminum chloride colorimetric method, according to the protocol of Singleton et al., (1999) with slight modifications using quercetin as a calibration standard [62]. Royal jelly (500 µL) was mixed with 100 µL of 10% aluminum chloride, 1.5 mL of methanol, 100 µL of 1M potassium acetate, and 2.8 mL of DI water. After that, the mixture was incubated in dark conditions for 30 min. The absorbance was then measured at 415 nm by a spectrophotometer (Thermo Scientific, Waltham, MA, USA). The total flavonoid content was calculated and reported in milligrams of quercetin equivalent per gram of royal jelly (mg QUE/g royal jelly).

### 4.13. High Performance Liquid Chromatography (HPLC) Analysis of Phytochemical Compound Content

The phytochemical compounds of royal jelly, including gallic acid and quercetin, were analyzed by the HPLC method [63]. The experiment was performed on the HPLC system (Agilent 1100 Series) with Agilent Eclipse XDB-C18, 5 µm size (150 × 4.6 mm) column and guard column, adjusted to 25 °C as th column temperature for separation. 50 mg/mL of royal jelly was dissolved in methanol and filtered through a 0.22 µm filter membrane. The prepared sample (20 µL) was then injected into the HPLC system. The mobile phase was a gradient mixture of mobile phase A (0.1% formic acid) and mobile phase B (methanol). The separation gradient was used at a ratio (%v/v) of A and B at 50:50 (0 min), 45:55 (5 min), 40:60 (10 min), 35:65 (15 min), 30:70 (20 min), 25:75 (25 min), 25:75 (30 min), 20:80 (35 min), 10:90 (40 min) and 0:100 (45 min). The HPLC system was adjusted to a flow rate of 1.0 mL/min, a running time of 60 min, and a UV detection of 267 nm. The standard curve was constructed with five concentrations of gallic acid (25.00, 12.50, 3.13, 1.56 µg/mL) and quercetin (50.00, 25.00, 12.50, 3.13 µg/mL). The area peak was compared to the standard curve of each compound to calculate the content of gallic acid and quercetin.

### 4.14. Statistical Analysis

The values expressed are means of three replicates of determination ± standard deviation (SD) and analyzed by One-Way Analysis of Variance (ANOVA). The data were evaluated by the statistical program in the SPSS Version 24.0 program (IBM, Chicago, IL, USA) with the significant difference at *p* < 0.05.

## 5. Conclusions

This study demonstrates that royal jelly has the ability to inhibit skin pathogenic bacteria, including *C. acnes*, MRSA, *P. aeruginosa*, *S. aureus*, *S. epidermidis,* and *Corynebacterium* spp. According to the bacterial cell membrane structures, royal jelly showed high antibacterial activity against Gram-positive bacteria, while its effectiveness decreased against Gram-negative bacteria. It was especially effective against MRSA, a well-known drug-resistant bacteria. Additionally, royal jelly has an anti-inflammatory effect on RAW264.7 macrophage cells stimulated with LPS. During this study, the total phenolic and flavonoid contents indicated that the antioxidant activity of royal jelly is due to the presence of phytochemical compounds. Royal jelly has strong antibacterial, anti-inflammatory, and antioxidant activities, making it an ideal component in skin care products and cosmetics, and can open up possibilities for future product development. Understanding the properties of royal jelly will improve its clinical and pharmaceutical uses in new and alternative therapies in the future.

## Figures and Tables

**Table 1:** Antibacterial activity of royal jelly samples against skin pathogenic bacteria by an agar well diffusion assay.

Royal JellySamples	Inhibition Zone Diameter (mm) *					
	Tested Bacteria					
	*Corynebacterium* spp.	*C. acnes*	MRSA	*P. aeruginosa*	*S. aureus*	*S. epidermidis*
RJ-LP1	23.67 ± 3.21	12.33 ± 0.58	15.00 ± 1.73	9.33 ± 0.58	13.67 ± 1.15	17.00 ± 2.00
RJ-CM1	19.33 ± 1.15	11.67 ± 0.58	13.67 ± 1.15	0	13.00 ± 1.00	14.00 ± 2.65
RJ-CM2	27.67 ± 2.31	13.33 ± 0.58	14.67 ± 0.58	10.33 ± 0.58	15.33 ± 1.53	16.33 ± 2.31
RJ-CM3	17.00 ± 1.73	14.67 ± 1.53	10.67 ± 0.58	10.00 ± 1.00	11.33 ± 1.15	10.33 ± 0.58
RJ-CM4	19.67 ± 0.58	15.00 ± 1.73	13.00 ± 1.00	10.33 ± 0.58	13.33 ± 1.53	11.67 ± 0.58
RJ-CM5	17.33 ± 0.58	0	12.33 ± 0.58	0	9.67 ± 0.58	15.67 ± 1.15
RJ-CM6	18.00 ± 1.00	0	10.33 ± 0.58	0	10.00 ± 0.00	11.67 ± 0.58
RJ-CM7	18.67 ± 0.58	13.00 ± 1.00	11.67 ± 0.58	0	11.67 ± 0.58	11.33 ± 0.58
RJ-CM8	17.33 ± 0.58	0	10.33 ± 0.58	0	11.67 ± 0.58	10.67 ± 0.58
**Control**						
Gentamicin	34.83 ± 0.29	42.17 ± 1.61	ND	31.17 ± 0.29	31.00 ± 1.32	30.67 ± 0.58
Vancomycin	ND	ND	31.33 ± 0.58	ND	ND	ND
Distilled water	0	0	0	0	0	0

* The data represent mean values of three replicates ± SD. ND: Not determined. RJ-LP: Royal jelly from Lamphun province, Thailand. RJ-CM: Royal jelly from Chiang Mai province, Thailand.

**Table 2:** Minimum inhibitory concentration (MIC) and minimum bactericidal concentration (MBC) of the royal jelly samples against skin pathogenic bacteria.

Royal JellySamples	MIC and MBC (mg/mL) *											
	Tested Bacteria											
	*Corynebacterium* spp.		*C. acnes*		MRSA		*P. aeruginosa*		*S. aureus*		*S. epidermidis*	
	MIC	MBC	MIC	MBC	MIC	MBC	MIC	MBC	MIC	MBC	MIC	MBC
RJ-LP1	18.75	18.75	18.75	37.50	37.50	37.50	75.00	75.00	37.50	75.00	75.00	75.00
RJ-CM1	18.75	18.75	37.50	37.50	37.50	75.00	75.00	75.00	37.50	75.00	75.00	75.00
RJ-CM2	18.75	37.50	75.00	75.00	37.50	37.50	75.00	75.00	37.50	75.00	75.00	150.00
RJ-CM3	18.75	37.50	75.00	150.00	37.50	75.00	75.00	150.00	37.50	150.00	75.00	150.00
RJ-CM4	18.75	37.50	75.00	75.00	37.50	37.50	75.00	75.00	37.50	75.00	37.50	75.00
RJ-CM5	18.75	18.75	37.50	37.50	37.50	37.50	75.00	75.00	75.00	75.00	37.50	37.50
RJ-CM6	18.75	18.75	75.00	75.00	75.00	150.00	150.00	150.00	75.00	75.00	75.00	75.00
RJ-CM7	18.75	18.75	37.50	37.50	75.00	75.00	75.00	150.00	75.00	75.00	75.00	75.00
RJ-CM8	18.75	18.75	75.00	75.00	75.00	75.00	150.00	150.00	75.00	75.00	75.00	75.00
**Control**												
Gentamicin	0.0039	0.0039	0.0039	0.0039	ND	ND	0.0039	0.0039	0.0078	0.0156	0.0039	0.0078
Vancomycin	ND	ND	ND	ND	0.0020	0.0020	ND	ND	ND	ND	ND	ND

* The data represent mean values of three replicates ± SD. ND: Not determined. RJ-LP: Royal jelly from Lamphun province, Thailand. RJ-CM: Royal jelly from Chiang Mai province, Thailand.

**Table 3:** IC_50_ of royal jelly samples on NO production of RAW264.7 cells.

Royal Jelly Samples	IC_50_ (mg/mL) *	
	Before Stimulation with LPS	After Stimulation with LPS
RJ-LP1	15.99 ± 1.52	9.23± 2.67
RJ-CM1	5.83 ± 1.49	4.46 ± 0.92
RJ-CM2	>20.00	9.37 ± 1.07
RJ-CM3	>20.00	16.67 ± 3.73
RJ-CM4	>20.00	15.19 ± 2.74
RJ-CM5	>20.00	13.31 ± 1.32
RJ-CM6	>20.00	16.37 ± 2.16
RJ-CM7	>20.00	15.08 ± 0.97
RJ-CM8	>20.00	16.35 ± 2.19

* The data represent mean values of three replicates ± SD. RJ-LP: Royal jelly from Lamphun province, Thailand. RJ-CM: Royal jelly from Chiang Mai province, Thailand.

**Table 4:** Antioxidant activity, total phenolic, and total flavonoid content of the royal jelly samples.

Royal Jelly Samples	Antioxidant Activity (mg TEAC/g Royal Jelly)	Total PhenolicContent (mg GAE/g Royal Jelly)	Total FlavonoidContent (mg QUE/g Royal Jelly)
RJ-LP1	2.03 ± 0.35 ^b^	6.64 ± 1.88 ^b^	4.93 ± 0.45 ^b^
RJ-CM1	4.13 ± 1.89 ^a^	8.61 ± 1.57 ^a^	6.25 ± 0.59 ^a^
RJ-CM2	3.61 ± 0.47 ^a^	7.27 ± 2.99 ^ab^	5.13 ± 0.86 ^b^
RJ-CM3	1.52 ± 0.08 ^b^	2.69 ± 0.17 ^c^	0.70 ± 0.15 ^c^
RJ-CM4	1.35 ± 0.11 ^b^	2.53 ± 0.25 ^c^	0.50 ± 0.11 ^c^
RJ-CM5	1.25 ± 0.18 ^b^	2.65 ± 0.42 ^c^	0.71 ± 0.06 ^c^
RJ-CM6	0.97 ± 0.23 ^b^	1.87 ± 0.26 ^c^	0.29 ± 0.10 ^c^
RJ-CM7	0.89 ± 0.14 ^b^	1.82 ± 0.38 ^c^	0.31 ± 0.04 ^c^
RJ-CM8	0.94 ± 0.17 ^b^	2.13 ± 0.56 ^c^	0.28 ± 0.09 ^c^

The data represent mean values of three replicates ± SD. Different subscript letters (a, b, c) indicate significant difference according to One-way ANOVA with Duncan’s multiple range tests at *p* < 0.05. RJ-LP: Royal jelly from Lamphun province, Thailand. RJ-CM: Royal jelly from Chiang Mai province, Thailand.

## Data Availability

The data presented in this study are available on request from the corresponding author.

## Supplementary Materials

The following supporting information can be downloaded at: https://www.mdpi.com/article/10.3390/molecules28030996/s1, Figure S1: Cytotoxicity of royal jelly RJ-LP1 (A), RJ-CM1 (B), RJ-CM2 (C), RJ-CM3 (D), RJ-CM4 (E), RJ-CM5 (F), RJ-CM6 (G), RJ-CM7 (H) and RJ-CM8 (I) on RAW264.7 cells. Table S1: Oligonucleotide primers used for qRT-PCR.
