# Supplementary material for: Inhibition of Skin Pathogenic Bacteria, Antioxidant and Anti-Inflammatory Activity of Royal Jelly from Northern Thailand
Source: Molecules. 2023 Jan 19;28(3):996. doi: 10.3390/molecules28030996 (PMC9920569; doi:10.3390/molecules28030996)
Supplement: Supplementary file 1 [file molecules-28-00996-s001.zip › molecules-2158529-supplementary.pdf]

Supplementary Materials

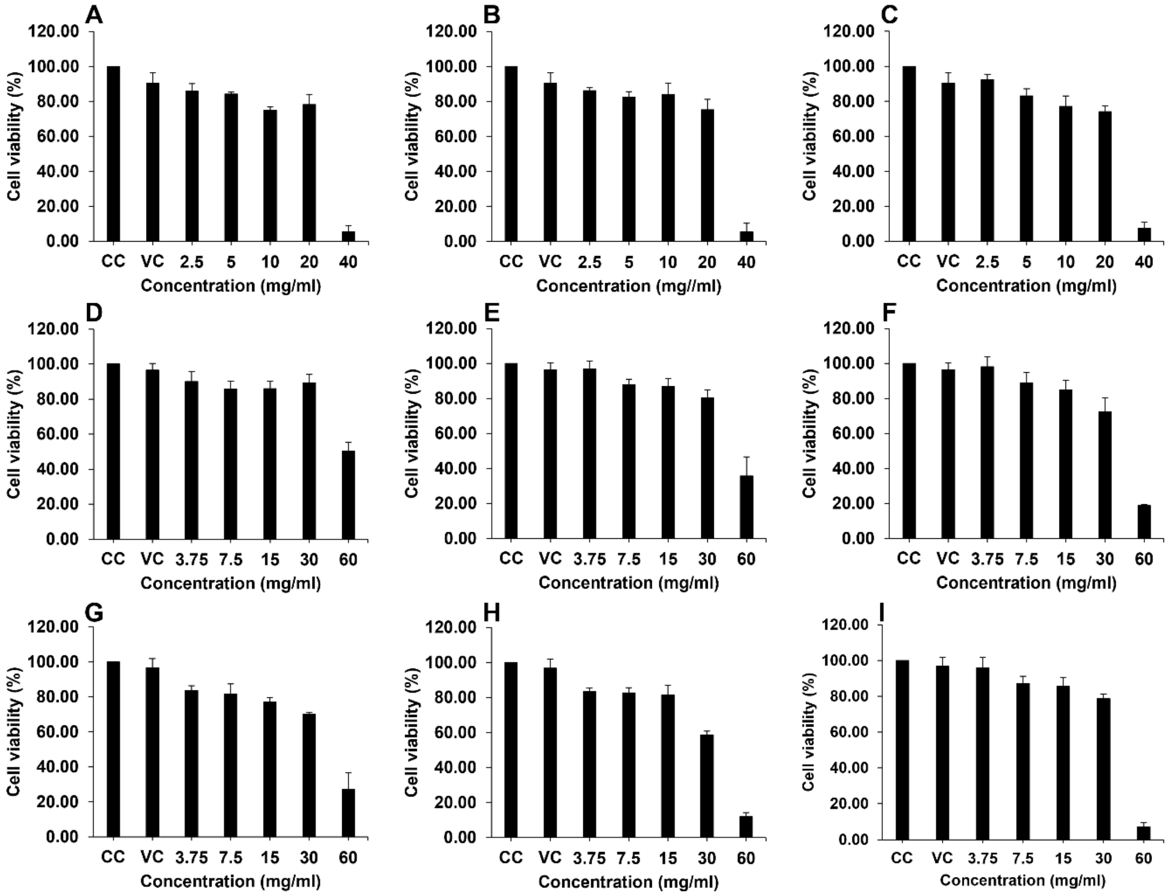

Figure S1. Cytotoxicity of royal jelly RJ-LP1 (A), RJ-CM1 (B), RJ-CM2 (C), RJ-CM3 (D), RJ-CM4 (E), RJ-CM5 (F), RJ-CM6 (G), RJ-CM7 (H) and RJ-CM8 (I) on RAW264.7 cells. Cell viability was measured using the MTT assay and calculated for percentage of cell viability comparing with the cell control (CC), and DMSO was used as a vehicle control (VC). The results are presented as mean ± SD values of triplicate independent experiments.

Table S1. Oligonucleotide primers used for qRT-PCR.

Genes		Sequences	References
iNOS	Forward	5′ TTC CAG AAT CCC TGG ACA AGC 3′	[58]
	Reverse	5′ TGG TCA AAC TCT TGG GGT TCG 3′	
COX-2	Forward	5′ AGA AGG AAA TGG CTG CAG AA 3′	[58]
	Reverse	5′ GCT CGG CTT CCA GTA TTG AG 3′	
IL-6	Forward	5′ GCT GGA GTC ACA GAA GGA GTG 3′	[59]
	Reverse	5′ GCA TAA CGC ACT AGG TTT GCC 3′	
ß-actin	Forward	5′ TGC TGT CCC TGT ATG CCT CTG 3′	[60]
	Reverse	5′ CTG TAG CCA CGC TCG GTC A 3′	
